# Supplementary figures and images for: Processing and Subcellular Localization of the Hepatitis E Virus Replicase: Identification of Candidate Viral Factories
Source: Front Microbiol. 2022 Feb 24;13:828636. doi: 10.3389/fmicb.2022.828636 (PMC8908324; doi:10.3389/fmicb.2022.828636)

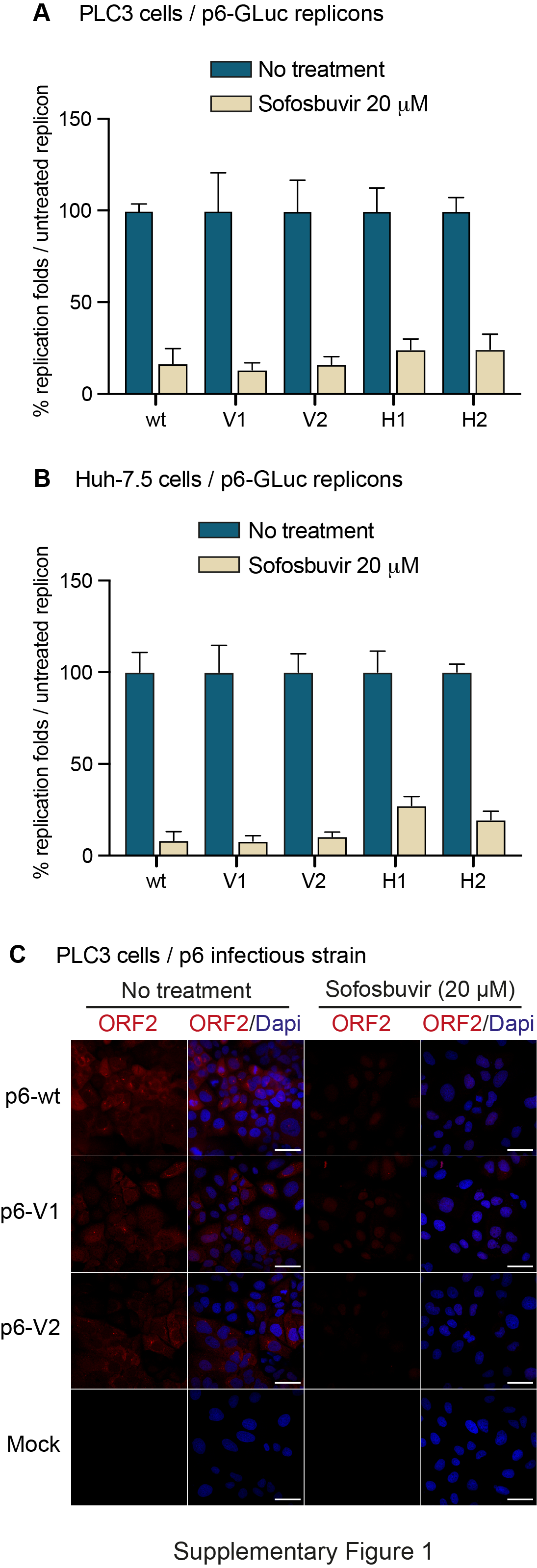

Supplement: Supplementary file 1 [file Image_1.TIF]

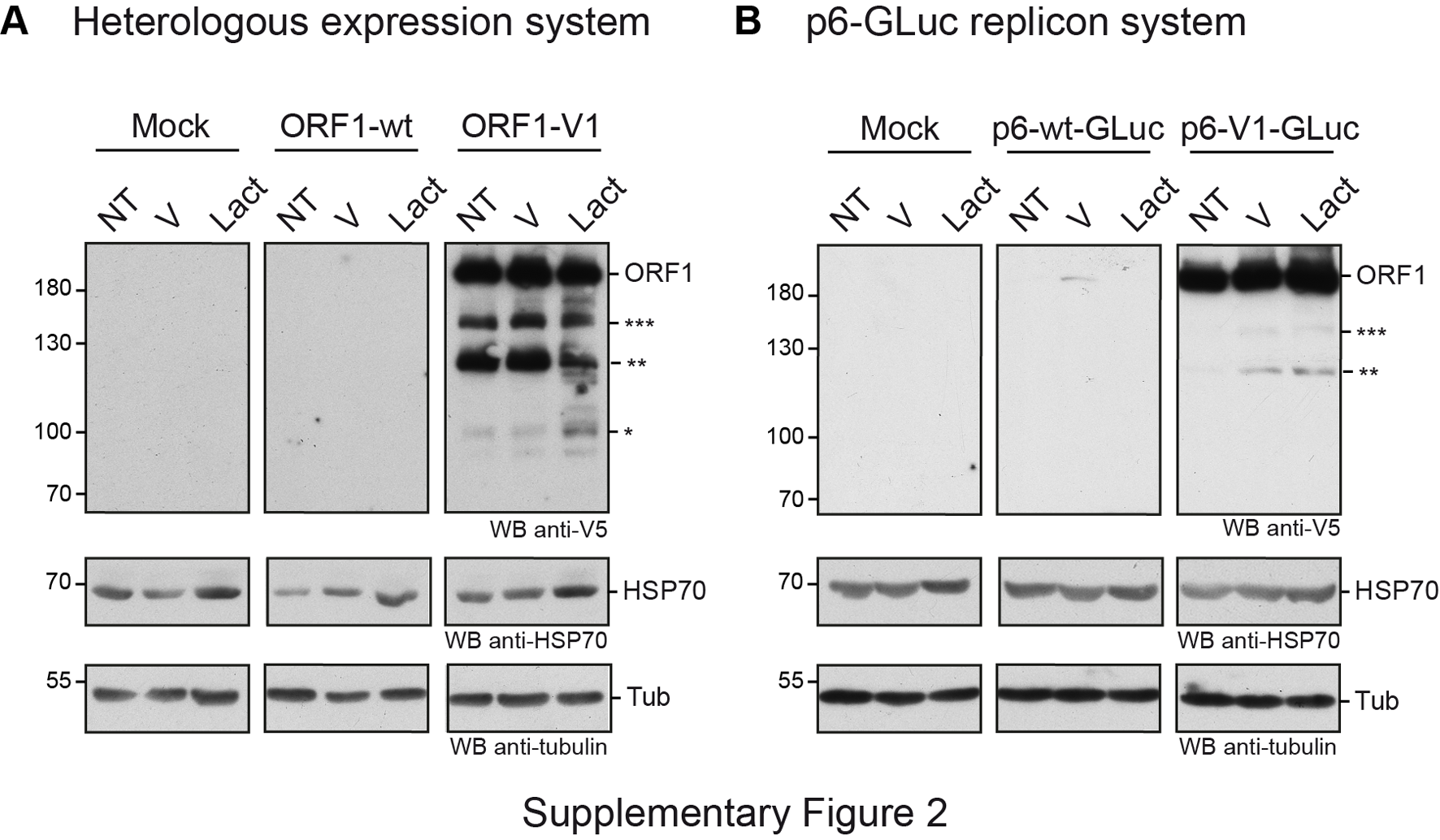

Supplement: Supplementary file 2 [file Image_2.TIF]

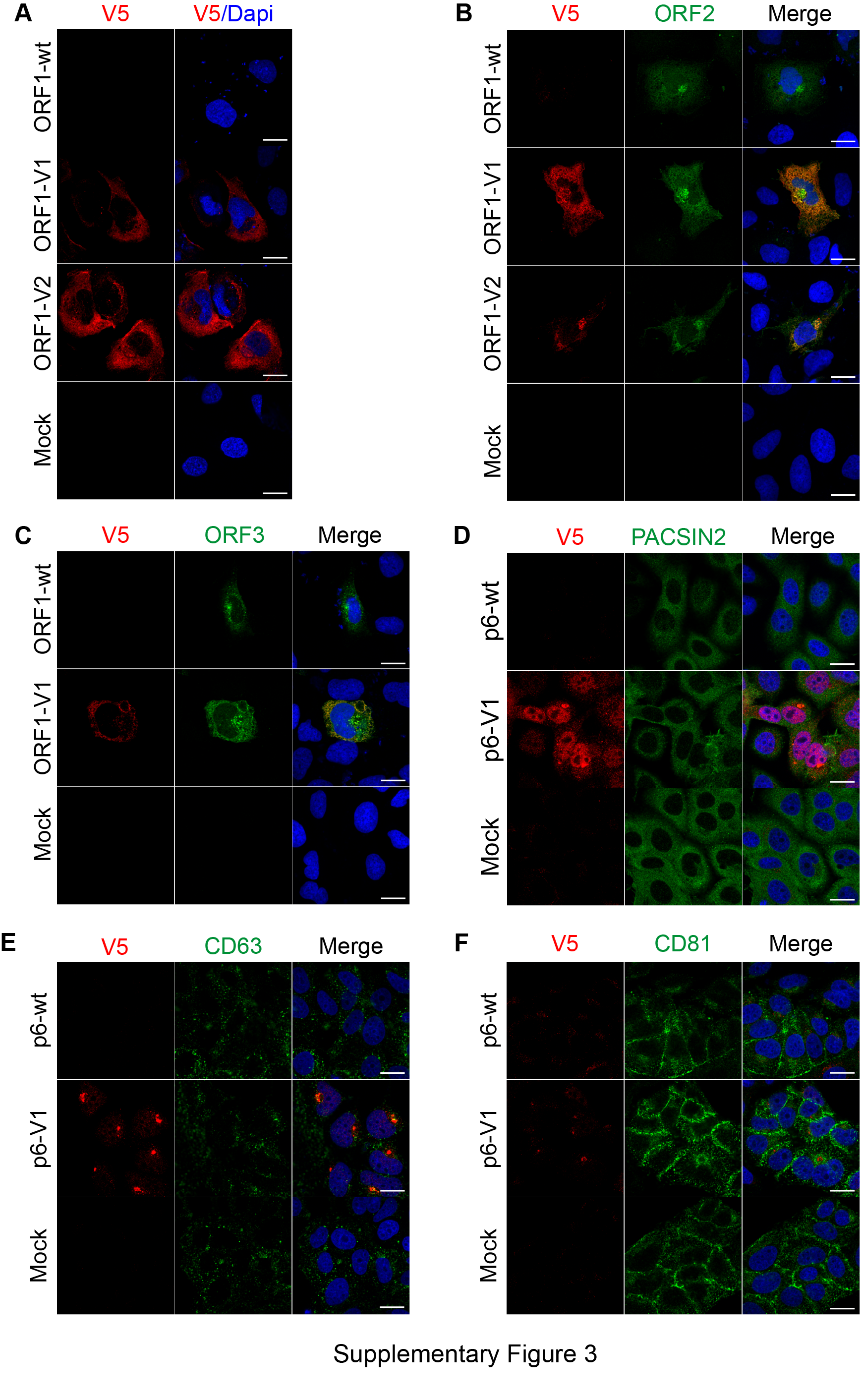

Supplement: Supplementary file 3 [file Image_3.TIF]
